# Meeting WHO physical activity standards may promote greater gut microbiota diversity and preservation of *Ruminococcus* in community-dwelling older women

**DOI:** 10.1265/ehpm.25-00335

**Published:** 2026-02-14

**Authors:** Mitsuru Shibata, Etsuko Muraki, Satoko Nezu, Katsuya Fujii, Satoshi Nobusako, Kayoko Maehara, Yumi Nakaya

**Affiliations:** 1 Department of Nutrition, Faculty of Health Sciences, Kio University, 4-2-2 Umaminaka, Koryo-cho, Kitakatsuragi-gun, Nara 635-0832, Japan; 2 Graduate School of Health Sciences, Kio University, 4-2-2 Umaminaka, Koryo-cho, Kitakatsuragi-gun, Nara 635-0832, Japan; 3 Department of Education, Faculty of Education, Kio University, 4-2-2 Umaminaka, Koryo-cho, Kitakatsuragi-gun, Nara 635-0832, Japan; 4 Neurorehabilitation Research Center, Kio University, 4-2-2 Umaminaka, Koryo-cho, Kitakatsuragi-gun, Nara 635-0832, Japan

**Keywords:** Gut microbiota, Physical activity, Older adults, *Ruminococcus*, Microbial diversity, Healthy aging

## Abstract

**Background:**

Gut microbiota plays a crucial role not only in digestion but also in systemic physiological functions, including immune and neural regulation. High microbial diversity contributes to intestinal homeostasis, whereas reduced diversity has been associated with various diseases. Physical activity is reported to influence both the composition and function of gut microbiota; however, the impact of daily physical activity on gut microbiota in older adults remains poorly understood. This study exploratorily investigated the association between objectively measured daily physical activity and gut microbial diversity and composition in older women who are at increased risk of reduced physical activity and gut microbial dysbiosis.

**Methods:**

A cross-sectional study assessed daily physical activity using an accelerometer-based activity monitor in 73 community-dwelling older women. We classified participants as meeting (n = 56) or not meeting (n = 17) the World Health Organization (WHO) physical activity guidelines. Fecal samples were collected and analyzed using 16S rRNA sequencing to evaluate gut microbial diversity and composition.

**Results:**

Participants not meeting the WHO activity guidelines exhibited lower gut microbial diversity (Observed Features: 134 ± 23 vs. 161 ± 45, Chao1 index: 137 ± 24 vs. 167 ± 49, all *p* < 0.05) and distinct microbial community structures (weighted UniFrac distances, PERMANOVA, *p* < 0.05) as compared with those meeting the guidelines. In particular, the relative abundance of short-chain fatty acid–producing *Ruminococcus* was reduced in the less active group.

**Conclusion:**

Habitual daily physical activity was associated with gut microbiota diversity and composition in older women in this exploratory study. In particular, the relative abundance of *Ruminococcus* may reflect differences in gut microbial function. Future longitudinal and interventional studies are needed to further clarify causal relationships and support the development of personalized strategies to promote gut health and healthy aging.

## Background

High gut microbial diversity in healthy adults plays a critical role in maintaining intestinal homeostasis. Conversely, reduced diversity has been associated with a broad spectrum of disease conditions, including inflammatory bowel disease, diabetes, obesity, and psychiatric disorders [1]. Historically, the gut microbiota was regarded mainly as a digestive aid, fermenting indigestible dietary fibers into short-chain fatty acids (SCFAs) and synthesizing vitamin K. Recently, evidence has highlighted its involvement in systemic physiological networks, particularly those governing neural and immune functions [2]. The therapeutic potential of fecal microbiota transplantation has further advanced conceptualization of the gut microbiota as a “third organ” [3]. Consequently, understanding gut microbiota has emerged as a pivotal approach for elucidating disease mechanisms and developing preventive and therapeutic strategies.

Diet, lifestyle behaviors, and pharmacological interventions are well-established determinants, and physical activity is emerging as an additional modifiable factor influencing both microbial composition and function [4]. Evidence from athletes suggests that physical activity enhances microbial diversity and modulates the abundance of specific bacterial taxa [5]. Despite this growing interest, few studies have examined the association between objectively measured physical activity and gut microbiota composition in the general population, particularly among community-dwelling older adults who are at increased risk of reduced physical activity and gut microbial dysbiosis.

Although research has begun to elucidate the connections between the gut microbiota, various organ systems, and disease processes, the relationship between daily physical activity and gut microbiota composition before disease onset remains poorly characterized in older adults. From a preventive medicine perspective, clarifying this association may inform the development of non-pharmacological interventions aimed at promoting healthy aging.

To address these questions, comprehensive profiling of the gut microbiota is essential. Traditional microbiological techniques have limited capacity to capture dominant anaerobes and their complex ecological interactions, but advances in next-generation sequencing (NGS) and bioinformatics now enable simultaneous identification of diverse microbial taxa, allowing for systems-level characterization of the gut microbiota [6, 7]. Leveraging these approaches, we hypothesized that higher levels of daily physical activity are associated with greater gut microbial diversity and distinct compositional profiles. To test this hypothesis, we objectively assessed physical activity using accelerometers rather than relying on subjective self-reports. Participants were stratified according to the World Health Organization (WHO) physical activity guidelines [8], which are based on evidence demonstrating a link between physical activity and reduced disease incidence and mortality. Subsequently, gut microbiota composition was analyzed to evaluate its association with physical activity levels.

## Methods

### Study design and participants

From November 2022 to July 2023, this cross-sectional study was conducted among 75 community-dwelling women aged 70 years or older, living independently in Nara Prefecture, Japan. On the first day, lifestyle and dietary surveys using a self-administered questionnaire method, physical measurements (height, weight, percent body fat, and grip strength), and interviews regarding medication and medical history were conducted. Participants were asked to wear an activity meter for seven consecutive days, starting the next day, and to perform a stool test within 7 days to measure gut microbiota. Individuals who were under outpatient treatment for gastrointestinal diseases, had a history of colon resection, or had taken antibiotics within the past 1 month were excluded. Individuals with other factors that might affect intestinal motility or gut microbiota, as evaluated by researchers, were also ineligible.

### Physical activity and sedentary behaviour

Physical activity was measured and evaluated using an Active Style Pro HJA-750C (OMURON, Kyoto, Japan) monitor equipped with a high-precision 3D acceleration sensor. Participants were instructed to wear the accelerometer on their waist (either side) continuously for one week, except during sleep, bathing, or swimming, while continuing their usual daily activities. They wore the device for as many waking hours as possible to maximize data completeness. Acceleration and intensity of physical activity were recorded; the accuracy of the intensity estimation has been validated with the Douglas bag method [9]. Non-wear time was defined as no acceleration signal for more than 60 consecutive minutes with allowance for up to 2 consecutive minutes of activities with intensity up to 1.0 METs [10, 11]. Data for participants with more than 600 minutes of data per day for more than 4 days were considered valid [12].

Physical activity was analyzed in 73 subjects who met the data adoption criteria. The time-sampling interval (epoch) was every minute during the 7 days. Intensity of physical activity was defined as sedentary behavior (≤1.5 METs) [13], low-intensity physical activity (LPA; 1.6–2.9 METs), and moderate-to-vigorous intensity physical activity (MVPA; ≥3.0 METs) [14]. In this study, a “sedentary bout” (SB) was defined as a continuous behavior measured from the start of a sitting behavior of ≤1.5 METs to the start of a physical activity of ≥1.6 METs. Additionally, an SB that lasted longer than 30 minutes was defined as a “prolonged sedentary bout” (PSB). A macro program (v.1.0) developed and distributed by Japan Physical Activity Research Platform was used to process the accelerometer data [15].

### Collection of fecal samples

Fecal samples were collected at home by participants using a fecal sampling kit (TechnoSuruga Laboratory Co., Ltd., Shizuoka, Japan) within 7 days after the survey and handed in within 14 days. Participants defecated on the collection sheet and sampled the feces with the collection brush; they then placed the brush in the container containing DNA preservation solution and shook it 5–6 times to mix the contents. Participants were instructed to keep the samples in a cool, dark place until the day of submission; the DNA is stable for one month at room temperature [16]. The samples were stored at 4 °C before DNA extraction.

### Gut microbiota analysis

DNA extraction and purification from fecal samples, PCR amplification of 16S rRNA gene, and sequencing using a next-generation sequencer were performed by TechnoSuruga Laboratory Co., Ltd. (Shizuoka, Japan). Microbial community structures were analyzed using Illumina NGS on the MiSeq platform (Illumina, San Diego, CA), as described previously [17]. Two-step polymerase chain reactions (PCRs) were performed on the purified DNA samples to obtain sequence libraries. The first PCR amplified the V3–V4 region of the 16S rRNA gene using primers Pro341F and Pro805R [17, 18]. The prepared libraries were subjected to sequencing of 300 paired-end bases using MiSeq Reagent Kit v3 (Illumina).

### 16S rRNA metagenome analysis

Quantitative Insights Into Microbial Ecology (QIIME) software package 2 (vv.2023.9) [19] was used for quality control and analysis of sequence reads. Raw sequenced amplicons were subjected to de-multiplexing based on unique barcodes assigned to each sample using q2-demux-plugin. Sequences with an average phred score of <30 were removed. Subsequent adapter and primer trimming, de-noising, merging overlapping paired-end reads, and removal of chimeric amplicons were performed with the DADA2 pipeline using q2-dada2-plugin [20]. Taxonomy was assigned to amplicon sequence variants (ASVs) using the q2-feature classifier classify-sklearn naïve Bayes taxonomy classifier against the SILVA database (SILVA release 138, 99%, with the qiime2 classifier trained on the 515F/806R V4 region of 16S). ASV sequences were aligned using Multiple Alignment using Fast Fourier Transform via q2-alignment, and a phylogenetic tree was constructed using FastTree2 via q2-phylogeny. Raw read counts were transformed into relative abundances by dividing each value by the total reads per sample and collapsed to taxonomic levels by summing their respective relative abundances.

Alpha and beta diversity statistics were generated via q2-core-metrics-phylogenetic. Alpha rarefaction was performed at the lowest sequencing depth (10,667) to avoid sequencing depth bias. Alpha diversity was measured using Chao 1, Observed Features, and Shannon and Simpson indices. Differences between alpha diversity indices were tested using the Kruskal–Wallis test (QIIME2). To estimate the similarity of microbial community structure between groups, beta diversity was quantitatively evaluated using Weighted UniFrac distance and Unweighted UniFrac distance metrics within QIIME2 and represented by a principal coordinate analysis (PCoA) plot. To assess the association between microbial community and groups, pairwise permutational multivariate analysis of variance (PERMANOVA) was implemented in QIIME2.

Microbiome Multivariable Associations with Linear Models 2 (MaAsLin2) is a multivariate statistical model that identifies associations between microbial features and metadata [21]. To assess differences in microbiome composition between groups based on physical activity, we fitted a general linear model for the abundance of microbial taxa using the default parameters of MaAsLin2. The associations’ *p*-values were corrected for multiple testing using Benjamini & Hochberg false discovery rate (FDR) control method. The threshold for *q*-values was set below 0.25 (default). The MaAsLin2 coefficient was interpreted as the effect size, indicating the strength and direction of the association between physical activity status and microbial features.

To predict gut microbiota function between groups, data analysis was performed through the Phylogenetic Investigation of Communities by Reconstruction of Unobserved States (PICRUSt2) pipeline via q2-picrust2-plugin [22–26]. PICRUSt2 outputs from the Kyoto Encyclopedia of Genes and Genomes (KEGG) database, release 70.0 [27], were analyzed and illustrated with Statistical Analysis of Metagenomic Profiles (STAMP) software, v.2.1.3 [28]. Within STAMP analysis, ANOVA followed by two-groups comparison was performed using White’s non-parametric t-test [29] with Storey FDR analysis [30].

### Dietary intake, physical measurement, and questionnaire survey

Dietary habits and nutrient intake levels were assessed using the brief-type self-administered diet history questionnaire (BDHQ). The BDHQ comprises 58 questions regarding food, beverage, and seasoning consumption during the preceding month to enable an estimation of total energy intake and micronutrient intake, and has been previously validated [31, 32]. Here, the obtained values were analyzed by adjusting them per 1,000 kcal of declared energy. Values with estimated energy intake exceeding 4,000 kcal were excluded due to suspected overreporting.

Height (cm), body mass (kg) and body fat percentage (%) were measured without footwear or outer clothes using a portable stadiometer (AD-6400; AND, Tokyo, Japan) and a bioelectrical impedance analysis device (TANITA TBF-535; Tanita, Tokyo, Japan). Handgrip strength was measured using a Smedley-type hand grip dynamometer (T.K.K.5401 Grip-D; Takei Scientific Instruments, Niigata, Japan). Two trials for each hand were performed; the highest value obtained was used for analysis. Background information such as age, living situation, and health status were collected through participant self-report. SARC-F, a screening tool for sarcopenia, was also used to help identify their health status.

### Classification of participants

According to the WHO Guidelines on physical activity and sedentary behaviour, older adults (≥65 years) should engage in 150–300 minutes of moderate-intensity aerobic activity per week, 75–150 minutes of vigorous-intensity aerobic activity per week, or an equivalent combination of moderate- and vigorous-intensity activity of comparable duration and intensity [8]. These recommendations were converted to daily equivalents for the present study: 21.4–42.9 minutes/day for moderate-intensity aerobic activity or 10.7–21.4 minutes/day for vigorous-intensity aerobic activity. Accordingly, cutoffs of 21.4 and 10.7 minutes/day were implemented for moderate and vigorous intensity, respectively.

No participants met the criterion for vigorous-intensity activity. Those who met the criterion for moderate-intensity activity were identical to those who met the combined moderate- and vigorous-intensity criterion. Therefore, we classified participants into those meeting the WHO physical activity recommendation (WHO-Met group) and those not meeting the recommendation (WHO-Unmet group).

### Statistical analysis

We compared participant characteristics, anthropometric measurements, handgrip strength, physical activity levels, lifestyle and health status, and nutrient and food group intakes between the two study groups. Descriptive statistics were calculated for all variables. Continuous variables are presented as median (interquartile range); categoric variables as number (percentage). For continuous variables, the Shapiro–Wilk test was used to assess normality. Normally distributed variables were analyzed using Student’s *t*-test; non-normally distributed variables were analyzed using Mann-Whitney *U* test. Categorical variables were compared using *χ*^2^ test or Fisher’s exact test, as appropriate. All statistical analyses were performed using SPSS Statistics for Windows v.25 (IBM Corp., Armonk, NY, USA). Statistical significance was set at *p* < 0.05 (two-tailed).

## Results

### Comparison of characteristics between the WHO-Met and WHO-unmet groups

We categorized participants into two groups according to the WHO 2020 Guidelines on physical activity and sedentary behavior: those who met the physical activity standard (WHO-Met group, n = 56), and those who did not (WHO-Unmet group, n = 17). Mean ages were 77.2 ± 4.8 years in the WHO-Unmet group and 75.5 ± 3.4 years in the WHO-Met group (Table 1).

**Table 1:** Participant characteristics and physical activities

	**WHO-Unmet (n = 17)**		**WHO-Met (n = 56)**		***p*-value**
Age (years)	78.0	(74.0–80.0)	74.5	(73.0–77.5)	0.173
BMI (kg/m^2^)	25.2	(21.8–27.2)	23.1	(20.2–25.0)	0.027
Handgrip strength (kg)	18.0	(16.0–20.0)	22.0	(19.5–24.6)	0.002
SARC-F (Point ≧4), n (%)	7	(41.2)	4	(7.1)	0.002
Number of steps (steps/day)	2513	(2079–3323)	4953	(3792–6100)	0.000
Employed, n (%)	15	(88.2)	49	(87.5)	0.652
Sitting work (workers only), n (%)	4	(26.7)	1	(2.1)	0.010
Activity limitation due to pain, n (%)	12	(70.6)	20	(35.7)	0.011
Percentage of SB time (%)	62.1	(59.4–65.7)	49.1	(40.9–54.4)	0.000
Percentage of LPA (%)	36.9	(33.4–38.5)	46.5	(39.3–52.8)	0.000
Percentage of MVPA (%)	1.4	(0.6–1.7)	5.6	(4.1–6.9)	0.000
Total PSB time (minutes/day)	245	(179.7–315.3)	179.1	(105.8–242.2)	0.002
Total PSB time/total sedentary behaviour time (%)	47.1	(34.7–56.1)	37.8	(28.9–45.4)	0.016

Data are expressed as median (interquartile range) or number (percentage).

WHO-Unmet: group not meeting WHO guidelines on physical activity and sedentary behaviour; WHO-Met: group meeting WHO guidelines; LPA: light-intensity physical activity; MVPA: moderate-to-vigorous physical activity; SB: sedentary bout (continuous sitting behavior); PSB: prolonged SB (>30 consecutive minutes).

As compared with the WHO-Met group, the WHO-Unmet group had lower handgrip strength and step count, higher BMI, higher percentages of participants with suspected sarcopenia, higher participants who refrained from activity due to musculoskeletal pain, and a higher percentage of PSB time during sedentary behavior time (Table 1). Additionally, the WHO-Unmet group had lower percentages of participants engaging in LPA and MVPA. There were no significant differences in energy or nutrient intake between the two groups (Table 2).

**Table 2:** Energy, nutrients and food groups intake for each group

	**WHO-Unmet (n = 17)**		**WHO-Met (n = 56)**		***p*-value**
Energy and Nutrients					
Total energy (kcal)	1630	(1439–1872)	1877	(1522–2261)	0.119
Proteins (g)	74.2	(68.0–89.0)	82.5	(64.6–102.3)	0.215
Lipids (g)	54.2	(46.0–66.1)	60.1	(50.0–74.7)	0.192
Carbohydrates (g)	215.2	(184.4–241.1)	243.4	(184.0–275.3)	0.422
Total dietary fiber (g)	12.8	(10.2–14.5)	13.3	(10.1–16.1)	0.827
Soluble dietary fiber (g)	3.3	(2.7–3.9)	3.5	(2.6–4.2)	0.755
Insoluble dietary fiber (g)	9.3	(7.2–10.5)	9.8	(7.2–11.7)	0.715
Sodium content (g)	9.7	(9.0–12.5)	11.7	(9.0–13.5)	0.776
Food Groups					
Cereals (g)	299.2	(198.4–376.8)	318.7	(216.9–407.1)	0.910
Tubers and roots (g)	40.0	(18.0–55.0)	50.0	(23.0–75.0)	0.234
Sugars (g)	3.6	(1.8–5.0)	3.7	(2.7–7.6)	0.330
Legumes (g)	60.3	(41.7–97.8)	68.6	(39.9–102.8)	0.915
Green and yellow vegetables (g)	105.6	(56.4–166.6)	102.7	(69.4–159.8)	0.806
Other vegetables (g)	137.2	(106.6–184.3)	167.2	(110.5–235.4)	0.422
Fruits (g)	128.1	(99.3–244.3)	150.7	(93.0–256.2)	0.989
Fish (g)	85.3	(54.3–125.6)	108.4	(61.3–158.1)	0.407
Meat (g)	63.6	(31.8–73.3)	68.6	(46.8–102.8)	0.138
Eggs (g)	47.1	(25.9–66.0)	47.1	(24.8–66.0)	0.642
Milk and dairy products (g)	120.0	(42.9–180.0)	165.0	(111.1–235.7)	0.097
Fats and oils (g)	8.6	(5.0–10.8)	10.2	(6.6–13.5)	0.187
Pastries (g)	50.0	(30.0–67.9)	45.7	(20.8–75.8)	0.555
Beverages (g)	620.0	(375.0–803.6)	626.6	(402.9–773.3)	0.958
Seasonings and spices (g)	159.3	(126.6–260.4)	161.6	(123.8–226.6)	0.591

Data are expressed as median (interquartile range) or number (percentage).

WHO-Unmet: group not meeting WHO guidelines on physical activity and sedentary behaviour; WHO-Met: group meeting WHO guidelines.

*One participant suspected of over-reporting was excluded.

### Lower gut microbiota diversity in the WHO-Unmet group

**Fig. 1:**
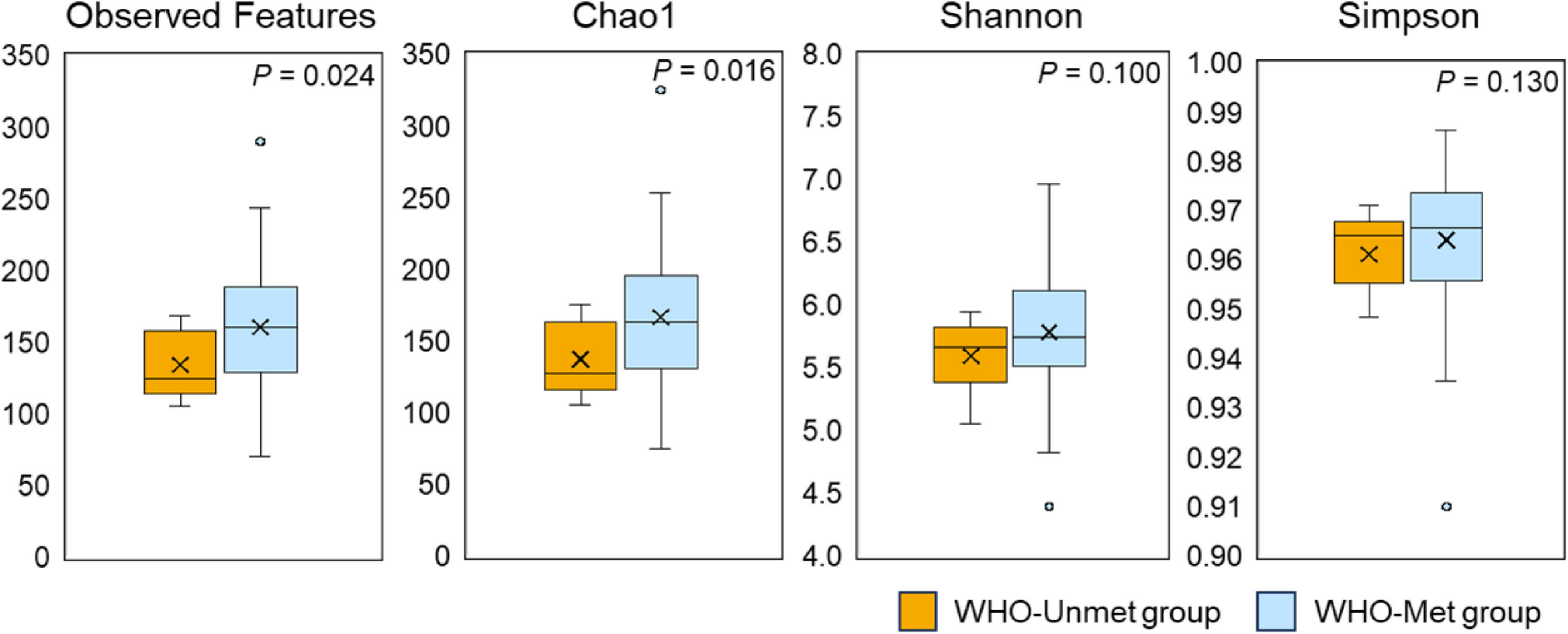
Alpha diversity in participants meeting versus not meeting the WHO physical activity guidelines We compared alpha diversity indices (Observed Features and Chao 1 indices for ASV richness estimation; Shannon and Simpson indices for ASV evenness estimation) using Mann-Whitney U test (*p* < 0.05).

Overall, we found that low physical activity was associated with lower gut microbiota diversity. Alpha diversity, which reflects microorganism diversity (primarily species richness and evenness) within a single sample was assessed by four indices. The Shannon and Simpson indices emphasize species evenness and dominance, while the Observed Features and Chao1 indices focus on species richness. Gut microbial richness was significantly lower in the WHO-Unmet group than in the WHO-Met group by the Observed Features (134 ± 23 vs. 161 ± 45) and Chao1 (137 ± 24 vs. 167 ± 49; Mann–Whitney U test, all *p* < 0.05) indices (Fig. 1). The Shannon and Simpson indices showed no differences between the two groups.

**Fig. 2:**
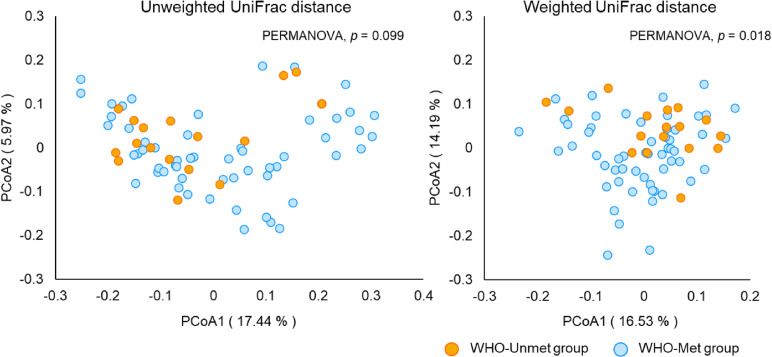
PCoA of gut microbiota in participants meeting versus not meeting the WHO physical activity guidelines Distances were calculated with unweighted and weighted UniFrac. PERMANOVA tests revealed that weighted distances between the WHO-Met and WHO-Unmet groups differed significantly (*p* < 0.05).

We assessed beta diversity, which reflects differences in microbial community composition, by using weighted and unweighted UniFrac distances to evaluate compositional dissimilarities of the gut microbiota between samples with different physical activity levels. PCoA based on weighted UniFrac distances revealed differences in microbial community structure between the WHO-Met and WHO-Unmet groups (PERMANOVA, R^2^ = 0.026, *p* = 0.018) (Fig. 2), indicating that the number of species (richness), their phylogenetic relationships, and relative abundances differed according to physical activity level.

### SCFA-producing *Ruminococcus* was reduced in the WHO-Unmet group

MaAsLin2 analysis identified four taxa with significant compositional differences between the WHO-Met group and the WHO-Unmet group (Table 3). We observed a significantly lower abundance of *Ruminococcus* (genus) (coef = −2.827, *q* = 0.004) and *Ruminococcus* (genus) spp. N/A (not applicable) (coef = −3.443, *q* = 0.054), and a higher abundance of *Clostridium glycyrrhizinilyticum* (coef = 0.788, *q* = 0.054) and *Blautia glucerasea* (coef = 1.109, *q* = 0.212) in the WHO-Unmet group as compared with the WHO-Met group.

**Table 3:** Significantly different taxa between participants meeting and not meeting the WHO physical activity guidelines

**Phylum**	**Class**	**Order**	**Family**	**Genus**	**Species**	**Increase/** **Decrease**	**Coefficient**	**Std Error**	***p*-value**	***q*-value**	**N not zero**
*Firmicutes*	*Clostridia*	*Oscillospirales*	*Ruminococcaceae*	*Ruminococcus*		−	−2.827	0.630	0.000	0.004	53
*Firmicutes*	*Clostridia*	*Oscillospirales*	*Ruminococcaceae*	*Ruminococcus*	*N*/*A*	−	−3.443	0.934	0.000	0.054	33
*Bacteroidota*	*Bacteroidia*					+	0.364	0.152	0.019	0.246	73
*Bacteroidota*						+	0.367	0.152	0.018	0.183	73
*Firmicutes*	*Clostridia*	*Lachnospirales*	*Lachnospiraceae*	*Clostridium*	*glycyrrhizinilyticum*	+	0.788	0.205	0.000	0.054	8
*Firmicutes*	*Clostridia*	*Lachnospirales*	*Lachnospiraceae*	*Blautia*	*glucerasea*	+	1.109	0.355	0.003	0.212	8

MaAsLin2 assessed multivariable correlations between groups and taxonomic abundance. All *p*-values were adjusted for false discovery rate (Benjamini-Hochberg, *q*-values); features with *q* < 0.25 were considered significant. WHO-Unmet: group not meeting WHO guidelines on physical activity and sedentary behaviour; WHO-Met: group meeting WHO guidelines; N/A: not applicable; “+”: higher relative abundance in WHO-Unmet group versus WHO-Met group; “−”: lower relative abundance.

### Butyric acid-producing taxa correlated with physical activity levels

Next, we assessed the correlation between variables such as age, handgrip strength, number of steps, LPA, MVPA, SB, PSB, and gut microbiota abundance using MaAsLin2 software (Table 4). Longer MVPA correlated with higher relative abundances of *Ruminococcus* (coef = 0.040, *q* = 0.038) and *Ruminococcus* (unclassified species) (coef = 0.054, *q* = 0.149). While longer SBs correlated with lower relative abundance of *Roseburia* (genus) (coef = −0.009, *q* = 0.098) and *Ruminococcaceae* (family) *Incertae Sedis* (coef = −0.006, *q* = 0.166). Finally, the longer PSB, the lower relative abundance of *Roseburia* (genus) (coef = −0.009, *q* = 0.244), *Turicibacter* (genus) (coef = −0.010, *q* = 0.169), and *Ruminococcaceae* (family) *Incertae Sedis* (coef = −0.007, *q* = 0.227).

**Table 4:** Gut microbiota taxa correlating with each variable using MaAsLin2 (*q*-value < 0.25)

**Variable**	**Phylum**	**Class**	**Order**	**Family**	**Genus**	**Species**	**Increase/** **Decrease**	**Coefficient**	**Std ** **Error**	***p*-value**	***q*-value**	**N not ** **zero**
Age	*Firmicutes*	*Clostridia*	*Oscillospirales*	*Oscillospiraceae*	*Flavonifractor*		−	−0.152	0.052	0.005	0.220	53
	*Firmicutes*	*Clostridia*	*Oscillospirales*	*Oscillospiraceae*	*Flavonifractor*	*N*/*A*	−	−0.154	0.052	0.004	0.249	53
	*Actinobacteriota*	*Actinobacteria*	*Micrococcales*	*Micrococcaceae*			+	0.174	0.050	0.001	0.025	14
	*Actinobacteriota*	*Actinobacteria*	*Micrococcales*	*Micrococcaceae*	*Rothia*		+	0.176	0.050	0.001	0.059	14
	*Actinobacteriota*	*Actinobacteria*	*Micrococcales*				+	0.176	0.052	0.001	0.041	14
	*Actinobacteriota*	*Actinobacteria*	*Micrococcales*	*Micrococcaceae*	*Rothia*	*N*/*A*	+	0.180	0.050	0.001	0.071	13
	*Firmicutes*	*Clostridia*	*Christensenellales*	*Christensenellaceae*	*Christensenellaceae_* *R.7_group*	*uncultured_* *Christensenella*	+	0.215	0.064	0.001	0.099	14
	*Firmicutes*	*Bacilli*	*Lactobacillales*	*Lactobacillaceae*	*Lactobacillus*	*N*/*A*	+	0.349	0.092	0.000	0.071	42
	*Firmicutes*	*Bacilli*	*Lactobacillales*	*Lactobacillaceae*			+	0.450	0.092	0.000	0.000	47
	*Firmicutes*	*Bacilli*	*Lactobacillales*	*Lactobacillaceae*	*Lactobacillus*		+	0.452	0.092	0.000	0.001	47

Handgrip strength	*Firmicutes*	*Bacilli*					−	−0.066	0.035	0.064	0.241	73
	*Firmicutes*	*Clostridia*					+	0.021	0.010	0.038	0.241	73
	*Bacteroidota*	*Bacteroidia*	*Bacteroidales*	*Barnesiellaceae*	*Coprobacter*	*Coprobacter_secundus*	+	0.110	0.034	0.002	0.206	9
	*Firmicutes*	*Clostridia*	*Lachnospirales*	*Lachnospiraceae*	*Roseburia*	*gut_metagenome*	+	0.112	0.036	0.003	0.233	16
	*Synergistota*	*Synergistia*					+	0.113	0.062	0.074	0.241	19
	*Desulfobacterota*						+	0.116	0.049	0.020	0.197	61
	*Desulfobacterota*	*Desulfovibrionia*					+	0.117	0.049	0.019	0.241	61
	*Firmicutes*	*Clostridia*	*Lachnospirales*	*Lachnospiraceae*	*Lachnoclostridium*		+	0.137	0.038	0.001	0.093	72
	*Firmicutes*	*Clostridia*	*Lachnospirales*	*Lachnospiraceae*	*Lachnoclostridium*	*metagenome*	+	0.142	0.043	0.002	0.206	42

Number of steps	*Proteobacteria*	*Gammaproteobacteria*	*Burkholderiales*	*Sutterellaceae*	*Sutterella*	*gut_metagenome*	+	0.000	0.000	0.000	0.092	10

LPA	*Firmicutes*	*Bacilli*					−	−0.004	0.002	0.013	0.083	73
	*Firmicutes*	*Clostridia*					+	0.001	0.000	0.010	0.083	73

MVPA	*Actinobacteriota*	*Actinobacteria*					+	0.026	0.010	0.013	0.170	73
	*Firmicutes*	*Clostridia*	*Oscillospirales*	*Ruminococcaceae*	*Ruminococcus*		+	0.040	0.010	0.000	0.038	53
	*Firmicutes*	*Clostridia*	*Oscillospirales*	*Ruminococcaceae*	*Ruminococcus*	*N*/*A*	+	0.054	0.015	0.001	0.149	33

SB	*Firmicutes*	*Clostridia*	*Oscillospirales*	*Ruminococcaceae*	*Incertae_Sedis*		−	−0.006	0.002	0.002	0.166	60
	*Firmicutes*	*Clostridia*	*Lachnospirales*	*Lachnospiraceae*	*Roseburia*		−	−0.009	0.003	0.001	0.098	61
	*Fusobacteriota*						+	0.007	0.003	0.025	0.245	19

PSB	*Firmicutes*	*Clostridia*	*Oscillospirales*	*Ruminococcaceae*	*Incertae_Sedis*		−	−0.007	0.002	0.003	0.227	60
	*Firmicutes*	*Clostridia*	*Lachnospirales*	*Lachnospiraceae*	*Roseburia*		−	−0.009	0.003	0.005	0.244	61
	*Firmicutes*	*Bacilli*	*Erysipelotrichales*	*Erysipelotrichaceae*	*Turicibacter*		−	−0.010	0.003	0.001	0.169	32
	*Fusobacteriota*						+	0.009	0.004	0.016	0.158	19
	*Fusobacteriota*	*Fusobacteriia*					+	0.009	0.004	0.016	0.206	19

The correlation between each variable and gut microbiota abundance was assessed using MaAsLin2. Indices were chosen at a univariate *q*-value (FDR-adjusted *p*-value) cutoff of 0.25. LPA: light-intensity physical activity; MVPA: moderate-to-vigorous physical activity; SB: sedentary bout (continuous sitting behavior); PSB: prolonged SB (>30 consecutive minutes); N/A: not applicable; “+”: higher relative abundance; “−”: lower relative abundance.

Lastly, taxa containing high proportions of well-known butyrate-producing bacteria, namely *Ruminococcaceae* (family), *Lachnospiraceae* (family), *Roseburia* (genus), and *Turicibacter* (genus), showed a positive correlation with MVPA and a negative correlation with sedentary behaviors such as SB and PSB.

### Butyric acid-producing *Lachnospiraceae* family correlated with dietary fiber intake

We also assessed the correlation between nutrients and gut microbiota abundance using MaAsLin2 (Table 5). Protein correlated with many taxa, and dietary fiber correlated with butyrate-producing bacteria from the *Lachnospiraceae* (family), but no taxa showed significant differences between the WHO-Met and WHO-Unmet groups. We observed no significant difference in nutrient intake between the two groups (Table 2), and the four taxa that differed significantly between the groups did not correlate with nutrient intake. Based on these observations, we considered the influence of diet on these differences to be minimal.

**Table 5:** Correlation between nutrients and gut microbiota

**Variable**	**Phylum**	**Class**	**Order**	**Family**	**Genus**	**Species**	**Increase/** **Decrease**	**Coefficient**	**Std Error**	***p*-value**	***q*-value**	**N not zero**
Proteins	*Firmicutes*	*Clostridia*	*Oscillospirales*	*Oscillospiraceae*	*uncultured*	*Intestinimonas_sp.*	−	−0.074	0.020	0.000	0.062	43
	*Firmicutes*	*Negativicutes*	*Acidaminococcales*	*Acidaminococcaceae*	*Phascolarctobacterium*	*N*/*A*	−	−0.133	0.046	0.005	0.161	47
	*Firmicutes*	*Clostridia*	*Lachnospirales*	*Lachnospiraceae*	*Eubacterium._hallii_group*	*N*/*A*	−	−0.146	0.043	0.001	0.088	53
	*Firmicutes*	*Clostridia*	*Christensenellales*	*Christensenellaceae*	*Christensenellaceae_* *R.7_group*	*uncultured_prokaryote*	+	0.053	0.018	0.004	0.161	21
	*Firmicutes*	*Clostridia*	*Lachnospirales*	*Lachnospiraceae*	*Lachnospiraceae_* *NK4A136_group*	*human_gut*	+	0.073	0.020	0.001	0.062	20
	*Firmicutes*	*Clostridia*	*Lachnospirales*	*Lachnospiraceae*	*Lachnospiraceae_* *NK4A136_group*		+	0.088	0.025	0.001	0.119	21
	*Firmicutes*	*Clostridia*	*Christensenellales*	*Christensenellaceae*	*Christensenellaceae_* *R.7_group*	*uncultured_Christensenella*	+	0.098	0.031	0.002	0.126	13
	*Firmicutes*	*Clostridia*	*Lachnospirales*	*Lachnospiraceae*	*Agathobacter*	*N*/*A*	+	0.120	0.040	0.003	0.161	40

Animal proteins	*Firmicutes*	*Clostridia*	*Oscillospirales*	*Oscillospiraceae*	*uncultured*	*Intestinimonas_sp.*	−	−0.074	0.019	0.000	0.039	43
	*Firmicutes*	*Negativicutes*	*Acidaminococcales*	*Acidaminococcaceae*	*Phascolarctobacterium*	*N*/*A*	−	−0.127	0.043	0.004	0.176	47
	*Firmicutes*	*Clostridia*	*Lachnospirales*	*Lachnospiraceae*	*Eubacterium._hallii_group*	*N*/*A*	−	−0.142	0.041	0.001	0.069	53
	*Firmicutes*	*Clostridia*	*Lachnospirales*	*Lachnospiraceae*	*Lachnospiraceae_* *NK4A136_group*	*human_gut*	+	0.069	0.019	0.001	0.065	20
	*Firmicutes*	*Clostridia*	*Lachnospirales*	*Lachnospiraceae*	*Lachnospiraceae_* *NK4A136_group*		+	0.082	0.024	0.001	0.146	21
	*Firmicutes*	*Negativicutes*	*Veillonellales.* *Selenomonadales*	*Veillonellaceae*	*Megasphaera*	*N*/*A*	+	0.091	0.030	0.003	0.155	12
	*Firmicutes*	*Clostridia*	*Christensenellales*	*Christensenellaceae*	*Christensenellaceae_* *R.7_group*	*uncultured_Christensenella*	+	0.093	0.029	0.002	0.128	13
	*Firmicutes*	*Clostridia*	*Oscillospirales*	*Ruminococcaceae*	*Ruminococcus*	*bicirculans*	+	0.099	0.036	0.007	0.200	39
	*Firmicutes*	*Clostridia*	*Lachnospirales*	*Lachnospiraceae*	*Agathobacter*	*N*/*A*	+	0.109	0.038	0.006	0.188	40

Vegetable proteins	*Firmicutes*	*Bacilli*	*Bacillales*	*Bacillaceae*	*Bacillus*	*N*/*A*	+	0.357	0.103	0.001	0.206	22
	*Firmicutes*	*Bacilli*	*Bacillales*	*Bacillaceae*			+	0.362	0.104	0.001	0.047	22
	*Firmicutes*	*Bacilli*	*Bacillales*	*Bacillaceae*	*Bacillus*		+	0.363	0.104	0.001	0.122	22
	*Firmicutes*	*Bacilli*	*Bacillales*				+	0.363	0.104	0.001	0.029	22

Animal lipids	*Bacteroidota*						−	−0.024	0.013	0.072	0.241	72
	*Actinobacteriota*						+	0.052	0.028	0.069	0.241	72
	*Desulfobacterota*						+	0.079	0.041	0.060	0.241	60
	*Actinobacteriota*	*Actinobacteria*					+	0.132	0.055	0.018	0.235	70

Vegetable lipids	*Firmicutes*	*Bacilli*					−	−0.089	0.038	0.023	0.211	72
	*Patescibacteria*	*Saccharimonadia*					+	0.057	0.026	0.033	0.211	11

Total dietary fiber	*Actinobacteriota*	*Coriobacteriia*	*Coriobacteriales*	*Eggerthellaceae*			−	−0.173	0.067	0.011	0.216	71
	*Firmicutes*	*Bacilli*	*Staphylococcales*	*Gemellaceae*			−	−0.174	0.067	0.011	0.216	13
	*Firmicutes*	*Clostridia*	*Oscillospirales*	*Ruminococcaceae*	*Incertae_Sedis*	*N*/*A*	−	−0.493	0.142	0.001	0.214	50
	*Firmicutes*	*Clostridia*	*Lachnospirales*	*Lachnospiraceae*			+	0.069	0.025	0.008	0.216	72

Soluble dietary fiber	*Firmicutes*	*Clostridia*	*Oscillospirales*	*Ruminococcaceae*	*Incertae_Sedis*	*N*/*A*	−	−1.807	0.527	0.001	0.242	50

Insoluble dietary fiber	*Firmicutes*	*Bacilli*	*Staphylococcales*	*Gemellaceae*			−	−0.254	0.097	0.011	0.250	13
	*Actinobacteriota*	*Coriobacteriia*	*Coriobacteriales*	*Eggerthellaceae*			−	−0.259	0.096	0.009	0.250	71
	*Firmicutes*	*Clostridia*	*Lachnospirales*	*Lachnospiraceae*			+	0.094	0.037	0.013	0.250	72

Ethanol	*Firmicutes*	*Bacilli*	*Erysipelotrichales*	*Erysipelotrichaceae*	*Holdemania*	*massiliensis*	+	0.062	0.020	0.003	0.206	12
	*Bacteroidota*	*Bacteroidia*	*Bacteroidales*	*Bacteroidaceae*	*Bacteroides*	*bacterium_NLAE.zl.H46*	+	0.068	0.017	0.000	0.038	8
	*Firmicutes*	*Clostridia*	*Lachnospirales*	*Lachnospiraceae*	*Roseburia*	*gut_metagenome*	+	0.092	0.027	0.001	0.112	15

Sodium content	*Firmicutes*	*Negativicutes*	*Veillonellales.* *Selenomonadales*	*Veillonellaceae*	*Dialister*	*N*/*A*	+	0.713	0.190	0.000	0.086	11

Correlation between nutrients and gut microbiota abundance assessed using MaAsLin2. Lipids and carbohydrates did not correlate with gut microbiota (results not shown). “+”: higher relative abundance; “−”: lower relative abundance.

### Differences in microbial community functions between the WHO-Unmet and WHO-Met groups

**Fig. 3:**
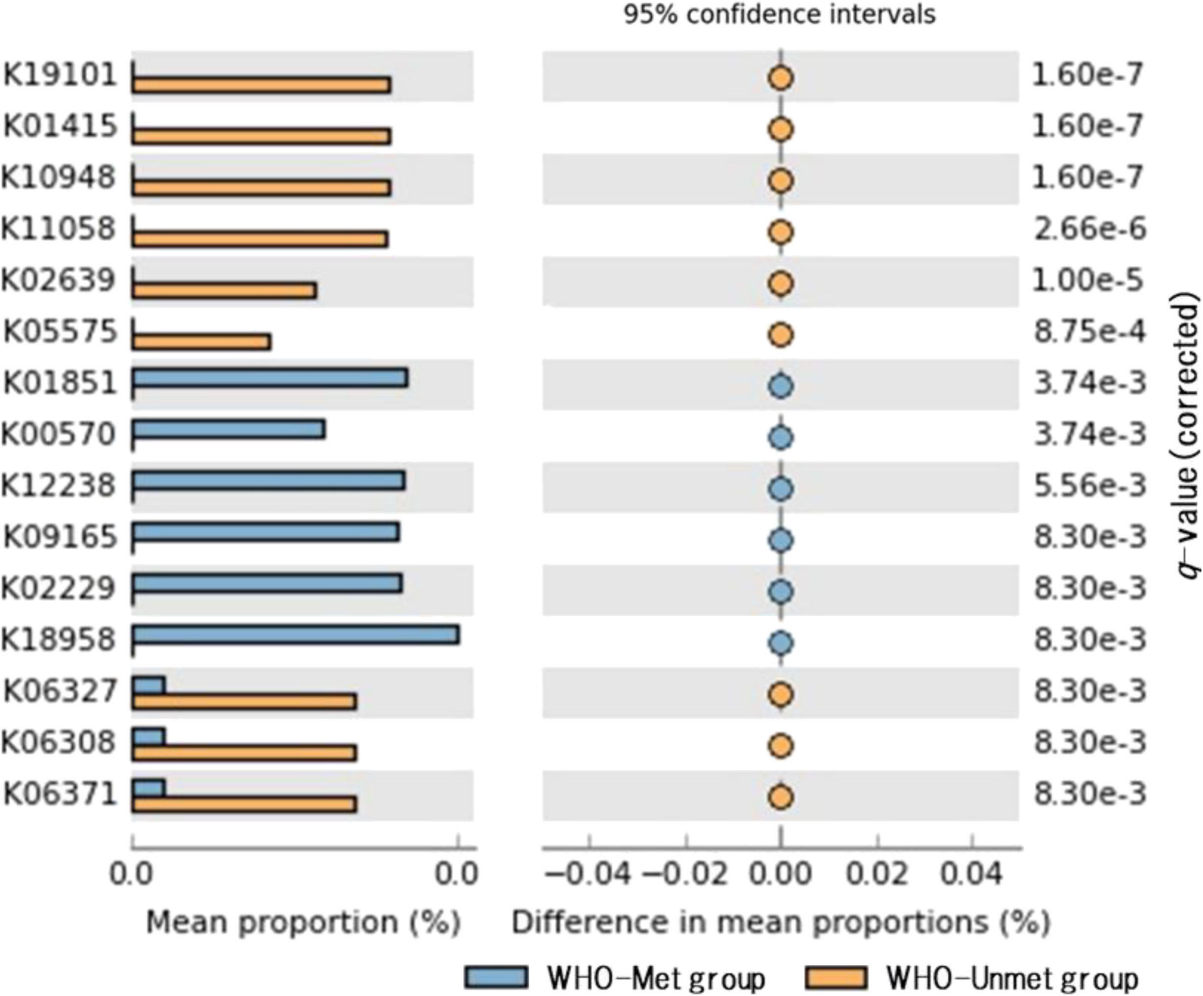
Potential differences in the functions of microbial communities between the WHO-Unmet and WHO-Met groups

We evaluated potential functional differences in the microbial communities between the WHO-Unmet and the WHO-Met groups using PICRUSt2 [22] (Fig. 3). In the WHO-Unmet group, predicted gene abundances were higher for β-lactam antibiotic resistance (K19101), endothelin-converting enzyme (K01415), bacterial toxins such as those from *Vibrio cholerae* and *Clostridium perfringens* (K10948, K11058), and proteins related to spore formation (K06327, K06308, K06371).

Genes predicted to be less abundant in the WHO-Unmet group included those involved in vitamin K_2_ biosynthesis (K01851) and glycerophospholipid metabolism (K00570). Among the genes related to glycerophospholipid metabolism, the predicted abundance of phosphatidylethanolamine *N*-methyltransferase (PEMT) was lower in the WHO-Unmet group.

## Discussion

Objectively measured daily physical activity was associated with gut microbiota diversity and composition in older women. Notably, participants not meeting the WHO physical activity guidelines showed a significant reduction in microbial diversity. Although alpha diversity analysis showed no significant differences between groups in the Shannon and Simpson indices, the Observed Features and Chao1 indices were significantly lower in the WHO-Unmet group. This suggests that older women with lower levels of physical activity may have a reduced number of gut bacterial species. Furthermore, beta diversity analysis revealed significant differences in microbial community structure according to physical activity status. These findings suggest a potential relationship between habitual physical activity and gut microbial diversity and composition, rather than demonstrating a direct causal influence.

Physical activity promotes intestinal peristalsis and shortens the transit time of intestinal contents, thereby exerting beneficial effects on the gut environment [33]. This process helps stabilize intestinal pH and suppress the accumulation of harmful metabolic byproducts, creating favorable conditions for beneficial bacteria to thrive. In contrast, reduced physical activity may lead to decreased intestinal motility, delayed gut transit, and disruption of the balance between fermentation and putrefaction in the gut. These changes may contribute to the overgrowth of specific bacterial taxa and a decline in microbial diversity. Moreover, physical exercise stimulates the secretion of myokines (e.g., interleukin-6, interleukin-10, and irisin) from skeletal muscles. These molecules possess anti-inflammatory properties and help to suppress systemic inflammation and maintain intestinal mucosal homeostasis [34]. Therefore, insufficient physical activity may lead to reduced myokine production, promoting chronic low-grade inflammation (so-called “inflammaging”) and compromising intestinal barrier function. Such an inflammatory environment may alter the gut microbiota composition, increasing the abundance of pro-inflammatory bacterial species and reducing beneficial microbes.

Significantly, we have demonstrated an association between objectively measured physical activity and gut microbial diversity in a general population of community-dwelling older women. Previous studies involving older adults have often relied on subjective instruments such as the International Physical Activity Questionnaire, which are susceptible to recall bias and overestimation. In contrast, we objectively assessed physical activity using accelerometers and categorized participants based on WHO physical activity guidelines. Many previous studies using accelerometers in older populations focused on men or mixed-age groups. Langsetmo et al. reported no significant association between physical activity and gut microbial diversity in older men [35]; similarly, Zhong et al. found no significant differences in alpha diversity associated with physical activity among older men and women [36]. In contrast, we have demonstrated an association between physical activity and gut microbial diversity in community-dwelling older women. Our detection of this association is mainly because physical activity was objectively measured using accelerometers and participants were clearly categorized based on the WHO guidelines. Furthermore, participants consisted of community-dwelling women in their mid-70s who remained relatively independent and physiologically responsive to lifestyle factors, which may have facilitated the detection of group-level differences in gut microbiota.

In younger populations, findings have been mixed. Bressa et al. found that physical exercise itself did not affect alpha diversity, but that sedentary parameters (i.e., sitting and resting time) were associated with microbial richness [37]. Among young adults, Castellanos et al. reported that sedentary individuals had lower alpha diversity relative to more active counterparts [38]. The observed reduction in alpha diversity among the WHO-Unmet group herein is consistent with these findings. While studies have reported a significant correlation between physical activity and alpha diversity of gut microbiota in young people, very few have reported this relationship in older adults. Clarke et al. reported that professional rugby players exhibited significantly higher alpha diversity relative to healthy non-athletes [39]. They suggested that more frequent and intense physical training may positively influence gut microbial alpha diversity. As their study targeted a specific trained population, however, its validity for the general older population is limited. In comparison, we demonstrated that habitual physical activity at levels consistent with WHO guidelines was associated with measurable differences in gut microbial diversity. This finding suggests broader applicability to general populations. Moreover, gut microbial diversity has been linked to various health outcomes, including metabolic, inflammatory, and neuropsychiatric disorders [1, 2]. Our findings support the notion that physical activity may contribute to health partly by modulating the gut microbiota.

Our MaAsLin2 analysis identified several bacterial taxa that differed significantly in relative abundance between the two groups. Notably, the relative abundance of genus *Ruminococcus*, which is involved in SCFA production, was lower in the WHO-Unmet group. *Ruminococcus* species act in the early steps of colonic fermentation, functioning as primary anaerobes that degrade dietary fiber and resistant starch to produce SCFAs such as acetate. SCFAs not only are an important energy source for intestinal epithelial cells but also exert anti-inflammatory effects and enhance muscle metabolism. In particular, butyrate-producing bacteria utilize acetate as a substrate, indicating metabolic “crosstalk” in which *Ruminococcus* contributes to the supply of acetate [40].

The observed reduction of *Ruminococcus* in older adults with low physical activity may reflect impaired saccharolytic function, which is essential for initiating the breakdown of complex carbohydrates, in the gut microbiome. This disruption could lead to reduced substrate availability for butyrate-producing genera such as *Faecalibacterium* and *Roseburia*, potentially resulting in decreased SCFA concentrations, compromised intestinal barrier function, and an environment more susceptible to inflammation.

The reduction in *Ruminococcus* may be associated with multiple factors linked to lower physical activity levels. First, less active individuals may consume fewer calories and less dietary fiber, creating an unfavorable environment for fiber-degrading bacteria such as *Ruminococcus*. However, there were no significant differences in habitual dietary fiber intake between the two groups. Second, physical activity-induced secretion of myokines may help maintain intestinal immunity and barrier integrity; a reduction in myokine levels could alter the gut environment and affect bacterial growth conditions. Third, decreased physical activity may disturb autonomic nervous system balance, potentially affecting gut motility and mucus secretion, thereby creating conditions less favorable for anaerobic bacteria including *Ruminococcus*. Lastly, reduced SCFA production may contribute to decreased muscle function through the gut-muscle axis, potentially reinforcing physical inactivity in a negative feedback loop.

Increased physical activity has been associated with a higher abundance of SCFA-producing bacteria, such as *Akkermansia*, *Faecalibacterium*, and *Ruminococcus* [41]. Among 100 community-dwelling older adults (mean age 69 years), whose physical activity was assessed using an accelerometer, the relative abundance of an operational taxonomic unit classified as *Ruminococcus* was negatively correlated with sedentary time and positively correlated with physical activity such as standing time [36], consistent with our findings. In contrast, the gut microbiota of older adults was found to exhibit a higher relative abundance of *Oscillospira* and *Ruminococcus* in individuals with physical frailty and sarcopenia than in those without [42], an observation that appears inconsistent with our findings. Sarcopenia is associated with chronic inflammation and reduced muscle-derived myokines in addition to physical activity; therefore, it may create a gut environment that favors the proliferation of specific *Ruminococcus* species, such as *R. gnavus*, which is resilient under inflammatory conditions.

Meanwhile, the taxa that were significantly more abundant in the WHO-Unmet group than in the WHO-Met group were *Clostridium glycyrrhizinilyticum* and *Blautia glucerasea*. *C. glycyrrhizinilyticum* is reported to hydrolyze glycyrrhizin, a compound derived from licorice root, into glycyrrhetinic acid, which has anti-inflammatory properties [43]. Although this bacterium belongs to *Clostridium* cluster XIVa, very few studies have investigated its characteristics or health relevance. Regarding *B. glucerasea*, Petrov et al. reported that its abundance decreases in patients with Parkinson’s disease [44], whereas Park et al. identified it as a predominant bacterium in Asian patients with depression [45]. In addition, *B. glucerasea* in the colon may convert dietary glucosylceramides into ceramides, which may help suppress local inflammation [46]. Therefore, it is plausible that in the WHO-Unmet group, these bacteria increased in a compensatory mechanism to counteract a loss of anti-inflammatory effects related to the reduced abundance of butyrate-producing bacteria. Future studies are warranted to elucidate the factors that contribute to the proliferation of these bacterial species and to clarify their potential roles in gut and systemic health.

Our functional prediction using PICRUSt2 suggested metabolic and functional changes in the gut microbiota of older women not meeting the recommended physical activity guidelines. Specifically, the predicted overrepresentation of β-lactam antibiotic resistance genes (K19101), toxin-associated genes related to pathogenic bacteria (K10948, K11058), and sporulation-associated genes (K06327, K06308, K06371) may suggest increased pathogenic potential within the gut environment. This observation supports the hypothesis that insufficient physical activity compromises intestinal homeostasis, potentially heightening susceptibility to inflammation and infection.

We also observed lower predicted abundances of the isomerase involved in vitamin K_2_ biosynthesis (K01851) and PEMT (K00570), a key enzyme in glycerophospholipid metabolism. Vitamin K_2_ contributes to mitochondrial function and insulin sensitivity in skeletal muscle via the SIRT1 signaling pathway, suggesting that its reduced biosynthesis may contribute to age-related muscle decline (sarcopenia) [47]. Likewise, the PEMT pathway is essential for generating phosphatidylcholine PEMT, a major constituent of the mitochondrial inner membrane, and its deficiency has been linked to mitochondrial structural defects and lipid metabolic abnormalities [48]. These predicted reductions in metabolic pathways relevant to energy production and cellular integrity are consistent with the molecular mechanisms underlying sarcopenia.

Our findings suggest that reduced physical activity may be associated with not only the taxonomic composition but also the functional potential of the gut microbiota, with possible downstream effects on host muscle metabolism and mitochondrial health. Such alterations support the concept of the gut–muscle axis, whereby the microbiome mediates the protective effects of physical activity on skeletal muscle function.

PICRUSt2 predictions are based on 16S rRNA gene data and do not directly reflect actual gene expression or metabolite concentrations. While this inherent limitation warrants cautious interpretation, the consistent group-level differences observed highlight the potential for physical activity to be associated with both structural and functional characteristics of the gut microbiota. Future studies employing shotgun metagenomics and metabolomics are essential to validate these predictions and to clarify the mechanistic links between physical activity, gut microbial function, and muscle health.

It is important to acknowledge several limitations of this exploratory study, particularly those related to generalizability. First, the number of participants in the group not meeting the WHO guidelines (n = 17) was smaller than that in the guideline-compliant group (n = 56). Because we divided participants based on physical activity measured by the 3D acceleration sensor, we were unable to control for this imbalance. It is desirable to adjust for confounding variables, such as BMI, musculoskeletal pain, and possible sarcopenia, which might affect gut microbiota, but multivariate analysis was not possible due to the sample size. Nevertheless, a sufficient number of sequencing reads were obtained from all participants, enabling the detection of significant differences in microbial diversity, composition, and predicted functions. Second, participants were recruited from a limited area within Nara Prefecture, Japan. Thus, the findings may reflect regional characteristics of a specific Japanese population, and caution is warranted when generalizing these results to other populations. Third, the cross-sectional design cannot demonstrate causal inference between physical activity and gut microbiota; therefore, longitudinal and intervention studies are required. Fourth, BDHQ, the dietary assessment method used, does not fully capture key components related to the gut microbiota, such as fermentable fiber, resistant starch, and fermented foods. Therefore, caution should be required to interpret the results obtained from the BDHQ. Fifth, we relied on 16S rRNA gene sequencing rather than metagenomic approaches, which restricts the ability to directly identify functional pathways. To address this, we employed PICRUSt2 to predict the metagenomic functional potential of microbial communities. However, studies using shotgun metagenomics and metabolomics will be important to provide more comprehensive insights regarding functional alterations in the microbiome potentially associated with physical activity. Finally, there remains the possibility of participants altering their habitual physical activity in response to wearing the activity monitors and seasonal variability; however, previous evidence suggests that such reactivity is unlikely to have substantially influenced the present findings [49].

A key strength of this study is the use of the Active Style Pro HJA-750C accelerometer, which enabled precise measurement of sedentary time, LPA, and MVPA using a single device. This comprehensive assessment allowed us to examine associations between each category of physical activity and both alpha and beta diversity of the gut microbiota.

## Conclusions

This exploratory cross-sectional study demonstrated that meeting the WHO physical activity guidelines was associated with the diversity and composition of the gut microbiota in community-dwelling older women. Notably, participants not meeting the guidelines exhibited reduced microbial richness and a lower relative abundance of the genus *Ruminococcus*, a key contributor to SCFA production. These findings suggest that habitual physical activity may help maintain intestinal homeostasis through gut function and immune regulation, potentially playing an important role in health maintenance in disease prevention in older adults.

Furthermore, maintaining regular daily physical activity—without structured exercise interventions—may support healthy longevity and help reduce the need for nursing care through its association with modulating the gut microbiota. Examining the relationship between physical activity and gut microbiota may also aid early screening for nursing care risk and the development of lifestyle-based preventive strategies. Specifically, *Ruminococcus* abundance correlated with physical activity, suggesting its potential as a biomarker of gut microbial functional integrity.

Future longitudinal and interventional studies are warranted to clarify the causal relationships between physical activity and the gut microbiota. Such research may provide a scientific foundation for personalized health support and preventive strategies against nursing care dependency through gut microbiome modulation.

## Abbreviations

WHO-Unmet: group not meeting WHO guidelines on physical activity and sedentary behaviour
WHO-Met: group meeting WHO guidelines on physical activity and sedentary behaviour
LPA: light-intensity physical activity
MVPA: moderate-to-vigorous physical activity
SB: sedentary bout (continuous sitting behavior)
PSB: prolonged sedentary bout (SB lasting >30 consecutive minutes)

## Declarations

### Availability of data and materials

Raw sequencing data in FASTQ format are publicly available from the NCBI Sequence Read Archive (SRA) under BioProject accession number PRJNA1311324 (https://www.ncbi.nlm.nih.gov/bioproject/PRJNA1311324), with corresponding BioSample accession numbers SAMN50881572–SAMN50881644. Library Strategy for all samples is Amplicon, targeting the 16S rRNA gene.

## References

[1] Lozupone CA, Stombaugh JI, Gordon JI, Jansson JK, Knight R. Diversity, stability and resilience of the human gut microbiota. Nature. 2012;489(7415):220–30. 22972295 10.1038/nature11550 PMC3577372

[2] Cryan JF, O’Riordan KJ, Cowan CSM, Sandhu KV, Bastiaanssen TFS, Boehme M, . The Microbiota-Gut-Brain Axis. Physiol Rev. 2019;99(4):1877–2013. 31460832 10.1152/physrev.00018.2018

[3] Khoruts A, Sadowsky MJ. Understanding the mechanisms of faecal microbiota transplantation. Nat Rev Gastroenterol Hepatol. 2016;13(9):508–16. 27329806 10.1038/nrgastro.2016.98 PMC5909819

[4] Mailing LJ, Allen JM, Buford TW, Fields CJ, Woods JA. Exercise and the Gut Microbiome: A Review of the Evidence, Potential Mechanisms, and Implications for Human Health. Exerc Sport Sci Rev. 2019;47(2):75–85. 30883471 10.1249/JES.0000000000000183

[5] Estaki M, Pither J, Baumeister P, Little JP, Gill SK, Ghosh S, . Cardiorespiratory fitness as a predictor of intestinal microbial diversity and distinct metagenomic functions. Microbiome. 2016;4(1):42. 27502158 10.1186/s40168-016-0189-7 PMC4976518

[6] Turnbaugh PJ, Ley RE, Hamady M, Fraser-Liggett CM, Knight R, Gordon JI. The human microbiome project. Nature. 2007;449(7164):804–10. 17943116 10.1038/nature06244 PMC3709439

[7] Qin J, Li R, Raes J, Arumugam M, Burgdorf KS, Manichanh C, . A human gut microbial gene catalogue established by metagenomic sequencing. Nature. 2010;464(7285):59–65. 20203603 10.1038/nature08821 PMC3779803

[8] WHO 2020 guidelines on physical activity and sedentary behaviour, 25 November 2020, https://www.who.int/publications/i/item/9789240015128.

[9] Ohkawara K, Oshima Y, Hikihara Y, Ishikawa-Takata K, Tabata I, Tanaka S. Real-time estimation of daily physical activity intensity by a triaxial accelerometer and a gravity-removal classification algorithm. Br J Nutr. 2011;105(11):1681–91. 21262061 10.1017/S0007114510005441

[10] Honda T, Chen S, Kishimoto H, Narazaki K, Kumagai S. Identifying associations between sedentary time and cardio-metabolic risk factors in working adults using objective and subjective measures: a cross-sectional analysis. BMC Public Health. 2014;14:1307. 25526746 10.1186/1471-2458-14-1307 PMC4302076

[11] Tudor-Locke C, Camhi SM, Troiano RP. A catalog of rules, variables, and definitions applied to accelerometer data in the National Health and Nutrition Examination Survey, 2003–2006. Prev Chronic Dis. 2012;9:E113. 22698174 10.5888/pcd9.110332 PMC3457743

[12] Trost SG, McIver KL, Pate RR. Conducting accelerometer-based activity assessments in field-based research. Med Sci Sports Exerc. 2005;37(11 Suppl):S531–43. 16294116 10.1249/01.mss.0000185657.86065.98

[13] Sedentary Behaviour Research Network. Letter to the editor: standardized use of the terms “sedentary” and “sedentary behaviours”. Appl Physiol Nutr Metab. 2012;37(3):540–2. 22540258 10.1139/h2012-024

[14] Haskell WL, Lee IM, Pate RR, Powell KE, Blair SN, Franklin BA, . Physical activity and public health: updated recommendation for adults from the American College of Sports Medicine and the American Heart Association. Med Sci Sports Exerc. 2007;39(8):1423–34. 17762377 10.1249/mss.0b013e3180616b27

[15] Japan Physical Activity Research Platform. http://paplatform.umin.jp (in Japanese).

[16] Hosomi K, Ohno H, Murakami H, Natsume-Kitatani Y, Tanisawa K, Hirata S, . Method for preparing DNA from feces in guanidine thiocyanate solution affects 16S rRNA-based profiling of human microbiota diversity. Sci Rep. 2017;7(1):4339. 28659635 10.1038/s41598-017-04511-0 PMC5489508

[17] Takahashi S, Tomita J, Nishioka K, Hisada T, Nishijima M. Development of a Prokaryotic Universal Primer for Simultaneous Analysis of Bacteria and Archaea Using Next-Generation Sequencing. PLoS One. 2014;9(8):e105592. 25144201 10.1371/journal.pone.0105592 PMC4140814

[18] Hisada T, Endoh K, Kuriki K. Inter- and intra-individual variations in seasonal and daily stabilities of the human gut microbiota in Japanese. Arch Microbiol. 2015;197(7):919–34. 26068535 10.1007/s00203-015-1125-0 PMC4536265

[19] Bolyen E, Rideout JR, Dillon MR, Bokulich NA, Abnet CC, Al-Ghalith GA, . Reproducible, interactive, scalable and extensible microbiome data science using QIIME 2. Nat Biotechnol. 2019;37(8):852–7. 31341288 10.1038/s41587-019-0209-9 PMC7015180

[20] Callahan BJ, McMurdie PJ, Rosen MJ, Han AW, Johnson AJ, Holmes SP. DADA2: High-resolution sample inference from Illumina amplicon data. Nat Methods. 2016;13(7):581–3. 27214047 10.1038/nmeth.3869 PMC4927377

[21] Mallick H, Rahnavard A, McIver LJ, Ma S, Zhang Y, Nguyen LH, . Multivariable association discovery in population-scale meta-omics studies. PLoS Comput Biol. 2021;17(11):e1009442. 34784344 10.1371/journal.pcbi.1009442 PMC8714082

[22] Douglas GM, Maffei VJ, Zaneveld JR, Yurgel SN, Brown JR, Taylor CM, . PICRUSt2 for prediction of metagenome functions. Nat Biotechnol. 2020;38(6):685–8. 32483366 10.1038/s41587-020-0548-6 PMC7365738

[23] Barbera P, Kozlov AM, Czech L, Morel B, Darriba D, Flouri T, . EPA-ng: massively parallel evolutionary placement of genetic sequences. Syst Biol. 2019;68(2):365–9. 30165689 10.1093/sysbio/syy054 PMC6368480

[24] Czech L, Barbera P, Stamatakis A. Genesis and Gappa: processing, analyzing and visualizing phylogenetic (placement) data. Bioinformatics. 2020;36(10):3263–5. 32016344 10.1093/bioinformatics/btaa070 PMC7214027

[25] Louca S, Doebeli M. Efficient comparative phylogenetics on large trees. Bioinformatics. 2018;34(6):1053–5. 29091997 10.1093/bioinformatics/btx701

[26] Ye Y, Doak TG. A parsimony approach to biological pathway reconstruction/inference for genomes and metagenomes. PLoS Comput Biol. 2009 Aug;5(8):e1000465. 19680427 10.1371/journal.pcbi.1000465 PMC2714467

[27] Kanehisa M, Goto S, Sato Y, Kawashima M, Furumichi M, Tanabe M. Data, information, knowledge and principle: back to metabolism in KEGG. Nucleic Acids Res. 2014;42(Database issue):D199–205. 24214961 10.1093/nar/gkt1076 PMC3965122

[28] Parks DH, Tyson GW, Hugenholtz P, Beiko RG. STAMP: statistical analysis of taxonomic and functional profiles. Bioinformatics. 2014;30(21):3123–4. 25061070 10.1093/bioinformatics/btu494 PMC4609014

[29] White JR, Nagarajan N, Pop M. Statistical methods for detecting differentially abundant features in clinical metagenomic samples. PLoS Comput Biol. 2009;5(4):e1000352. 19360128 10.1371/journal.pcbi.1000352 PMC2661018

[30] Storey JD, Tibshirani R. Statistical significance for genomewide studies. Proc Natl Acad Sci U S A. 2003;100(16):9440–5. 12883005 10.1073/pnas.1530509100 PMC170937

[31] Kobayashi S, Murakami K, Sasaki S, Okubo H, Hirota N, Notsu A, . Comparison of relative validity of food group intakes estimated by comprehensive and brief-type self-administered diet history questionnaires against 16 d dietary records in Japanese adults. Public Health Nutr. 2011;14(7):1200–11. 21477414 10.1017/S1368980011000504

[32] Kobayashi S, Honda S, Murakami K, Sasaki S, Okubo H, Hirota N, . Both comprehensive and brief self-administered diet history questionnaires satisfactorily rank nutrient intakes in Japanese adults. J Epidemiol. 2012;22(2):151–9. 22343326 10.2188/jea.JE20110075 PMC3798594

[33] Monda V, Villano I, Messina A, Valenzano A, Esposito T, Moscatelli F, . Exercise Modifies the Gut Microbiota with Positive Health Effects. Oxid Med Cell Longev. 2017;2017:3831972. 28357027 10.1155/2017/3831972 PMC5357536

[34] Lu Z, Wang Z, Zhang XA, Ning K. Myokines May Be the Answer to the Beneficial Immunomodulation of Tailored Exercise-A Narrative Review. Biomolecules. 2024;14(10):1205. 39456138 10.3390/biom14101205 PMC11506288

[35] Langsetmo L, Johnson A, Demmer RT, Fino N, Orwoll ES, Ensrud KE, . The Association between Objectively Measured Physical Activity and the Gut Microbiome among Older Community Dwelling Men. J Nutr Health Aging. 2019;23(6):538–46. 31233075 10.1007/s12603-019-1194-x PMC6618308

[36] Zhong X, Powell C, Phillips CM, Millar SR, Carson BP, Dowd KP, . The Influence of Different Physical Activity Behaviours on the Gut Microbiota of Older Irish Adults. J Nutr Health Aging. 2021;25(7):854–61. 34409962 10.1007/s12603-021-1630-6 PMC12280670

[37] Bressa C, Bailén-Andrino M, Pérez-Santiago J, González-Soltero R, Pérez M, Montalvo-Lominchar MG, . Differences in gut microbiota profile between women with active lifestyle and sedentary women. PLoS One. 2017;12(2):e0171352. 28187199 10.1371/journal.pone.0171352 PMC5302835

[38] Castellanos N, Diez GG, Antúnez-Almagro C, Bailén M, Bressa C, González Soltero R, . A Critical Mutualism - Competition Interplay Underlies the Loss of Microbial Diversity in Sedentary Lifestyle. Front Microbiol. 2020;10:3142. 32038575 10.3389/fmicb.2019.03142 PMC6987436

[39] Clarke SF, Murphy EF, O’Sullivan O, Lucey AJ, Humphreys M, Hogan A, . Exercise and associated dietary extremes impact on gut microbial diversity. Gut. 2014;63(12):1913–20. 25021423 10.1136/gutjnl-2013-306541

[40] Culp EJ, Goodman AL. Cross-feeding in the gut microbiome: Ecology and mechanisms. Cell Host Microbe. 2023;31(4):485–99. 37054671 10.1016/j.chom.2023.03.016 PMC10125260

[41] Dziewiecka H, Buttar HS, Kasperska A, Ostapiuk-Karolczuk J, Domagalska M, Cichoń J, Skarpańska-Stejnborn A. Physical activity induced alterations of gut microbiota in humans: a systematic review. BMC Sports Sci Med Rehabil. 2022 Jul 7;14(1):122. 35799284 10.1186/s13102-022-00513-2 PMC9264679

[42] Picca A, Ponziani FR, Calvani R, Marini F, Biancolillo A, Coelho-Junior HJ, . Gut Microbial, Inflammatory and Metabolic Signatures in Older People with Physical Frailty and Sarcopenia: Results from the BIOSPHERE Study. Nutrients. 2019;12(1):65. 31887978 10.3390/nu12010065 PMC7019826

[43] Sakuma K, Kitahara M, Kibe R, Sakamoto M, Benno Y. Clostridium glycyrrhizinilyticum sp. nov., a glycyrrhizin-hydrolysing bacterium isolated from human faeces. Microbiol Immunol. 2006;50(7):481–5. 16858139 10.1111/j.1348-0421.2006.tb03818.x

[44] Petrov VA, Saltykova IV, Zhukova IA, Alifirova VM, Zhukova NG, Dorofeeva YB, . Analysis of Gut Microbiota in Patients with Parkinson’s Disease. Bull Exp Biol Med. 2017;162(6):734–7. 28429209 10.1007/s10517-017-3700-7

[45] Park S, Li C, Wu X, Zhang T. Gut Microbiota Alterations and Their Functional Differences in Depression According to Enterotypes in Asian Individuals. Int J Mol Sci. 2023;24(17):13329. 37686135 10.3390/ijms241713329 PMC10487633

[46] Furuya H, Ide Y, Hamamoto M, Asanuma N, Hino T. Isolation of a novel bacterium, Blautia glucerasei sp. nov., hydrolyzing plant glucosylceramide to ceramide. Arch Microbiol. 2010;192(5):365–72. 20354843 10.1007/s00203-010-0566-8

[47] Su X, Wang W, Fang C, Ni C, Zhou J, Wang X, . Vitamin K2 Alleviates Insulin Resistance in Skeletal Muscle by Improving Mitochondrial Function Via SIRT1 Signaling. Antioxid Redox Signal. 2021 Jan 10;34(2):99–117. 32253917 10.1089/ars.2019.7908

[48] Calzada E, Onguka O, Claypool SM. Phosphatidylethanolamine Metabolism in Health and Disease. Int Rev Cell Mol Biol. 2016;321:29–88. 26811286 10.1016/bs.ircmb.2015.10.001 PMC4778737

[49] Powell C, Browne LD, Carson BP, Dowd KP, Perry IJ, Kearney PM, . Use of Compositional Data Analysis to Show Estimated Changes in Cardiometabolic Health by Reallocating Time to Light-Intensity Physical Activity in Older Adults. Sports Med. 2020;50(1):205–17. 31350674 10.1007/s40279-019-01153-2

